# Remote Monitoring of Critically-Ill Post-Surgical Patients: Lessons from a Biosensor Implementation Trial

**DOI:** 10.3390/healthcare9030343

**Published:** 2021-03-18

**Authors:** Mariana Restrepo, Ann Marie Huffenberger, C William Hanson, Michael Draugelis, Krzysztof Laudanski

**Affiliations:** 1 College of Arts and Sciences, University of Pennsylvania, Philadelphia, PA 19104, USA; rmariana@sas.upenn.edu; 2 Penn Medicine Center for Connected Care, Clinical Practices of the University of Pennsylvania, Philadelphia, PA 19104, USA; ann.huffenberger@pennmedicine.upenn.edu (A.M.H.); william.hanson@pennmedicine.upenn.edu (CW.H.III); 3 Department of Anesthesiology and Critical Care, University of Pennsylvania, Philadelphia, PA 19104, USA; 4 Department of Radiology, University of Pennsylvania Health System, Philadelphia, PA 19104, USA; michael.draugelis@pennmedicine.upenn.edu; 5 Department of Anesthesiology and Critical Care, Hospital of the University of Pennsylvania, Philadelphia, PA 19104, USA; 6 Department of Neurology, University of Pennsylvania, Philadelphia, PA 19104, USA; 7 Leonard Davis Institute of Health Economics, University of Pennsylvania, Philadelphia, PA 19104, USA

**Keywords:** wearable biosensors, critical care, vital sign monitoring, bio-monitoring system, technology acceptance, integration, implementation

## Abstract

Biosensors represent one of the numerous promising technologies envisioned to extend healthcare delivery. In perioperative care, the healthcare delivery system can use biosensors to remotely supervise patients who would otherwise be admitted to a hospital. This novel technology has gained a foothold in healthcare with significant acceleration due to the COVID-19 pandemic. However, few studies have attempted to narrate, or systematically analyze, the process of their implementation. We performed an observational study of biosensor implementation. The data accuracy provided by the commercially available biosensors was compared to those offered by standard clinical monitoring on patients admitted to the intensive care unit/perioperative unit. Surveys were also conducted to examine the acceptance of technology by patients and medical staff. We demonstrated a significant difference in vital signs between sensors and standard monitoring which was very dependent on the measured variables. Sensors seemed to integrate into the workflow relatively quickly, with almost no reported problems. The acceptance of the biosensors was high by patients and slightly less by nurses directly involved in the patients’ care. The staff forecast a broad implementation of biosensors in approximately three to five years, yet are eager to learn more about them. Reliability considerations proved particularly troublesome in our implementation trial. Careful evaluation of sensor readiness is most likely necessary prior to system-wide implementation by each hospital to assess for data accuracy and acceptance by the staff.

## 1. Introduction

The ability of biosensors to wirelessly, un-obstructively, and effortlessly monitor patients has become a fascinating prospect for healthcare [1]. They offer an opportunity to improve patient care while reducing costs and increasing patient and staff satisfaction [2,3]. At a minimum, most biosensors collect body temperature, pulse, heart rate variability, respiration rate, peripheral capillary oxygen saturation (SpO_2_), sleep, and movement. Although sensors can quite often deliver additional data, it is unclear if they can increase the effectiveness of healthcare delivery.

In order to effectively integrate biosensors into healthcare workflow, several factors have to be fulfilled [4]. Foremost, the reliability of the equipment needs to be assessed. A previous study found that when comparing SpO_2_ measurements between five types of biosensors and a clinical vital sign monitor, a range of 85–100% of biosensor measurements fell within three percentage points of the clinical monitor, depending on the type of biosensor [5,6]. However, the same study alternatively established that this range shifted to 93.5–100% of biosensor measurements falling within three beats per minute (BPM) of the clinical monitor [5]. It is also notable that mean skin temperature measured by biosensors can vary up to 2 °C from axillary measurements [7]. Furthermore, recordings from the research-grade biosensors proved less accurate than those intended for consumers [6,8,9]. Both the consistency and accuracy of some vital signs are much dependent on the device model [6,8,9]. Finally, the devices must take into account features specific to patients [10]. These inconsistencies across differing vital signs could introduce deceptive data trends that would undermine the feasibility of implementing biosensors in a critical care setting.

The implementation of biosensors in the workflow must be very well-planned and unit-specific [11]. The demands for perioperative care are particularly sensitive to interruption of the signal, while in other instances, accuracy may matter more. The data has to be delivered from sensors via a secure wireless network connection to provide a clear advantage over the existing infrastructure [4]. Establishing such a link securely and reliably is a complex task, especially in a hospital system with multiple entities operating off varying information system infrastructures [12]. Providing similar monitoring at home is even more complex. Acceptance of the sensor must be high across all parties involved [4,11,13,14]. Patients should value the sensor as an improvement over prior solutions. Sensors should be especially comfortable and undisruptive in perioperative settings. Providers should expect robust and reliable sets of data adding to the care being provided. Similarly, nursing staffs seek to ease the burden of continuously monitoring patients remotely, allowing biosensors to improve the quality and safety of patient care. All these requirements are particularly important for perioperative care, especially in in-home settings. A useful framework for the implementation of biosensors is provided by the ABCDEF bundle by suggesting a focus on which parameters yield most of the value [15]. Understanding potential barriers to this integration is the key to major transformations in healthcare [4,9,10,16].

This study describes the process of implementation of a multisensory biosensor platform to analyze up to 22 parameters and features in intensive care unit (ICU) patients. We aimed to describe our implementation process experiences, with special emphasis on comparing data streams from patients being monitored by biosensors versus standard hospital physiological monitoring. We also analyzed acceptance of the technology by patients, providers, and nurses. Past studies have found that while biosensors have extensive potential for real-world adaptations, functional challenges, including data validity and stability, need to be overcome first before defining practical applications [17].

## 2. Materials and Methods

The IRB at the University of Pennsylvania approved the study (#832633). Data were collected in 2020.

This is a pilot study testing the feasibility and robustness of the two types of wearable biosensors in anticipation of future deployment. One of the sensors is commercially available and used predominately for personal care, and it has not been previously tested in a healthcare ICU setting. The other one represented a biosensor that was developed and manufactured for healthcare use by a start-up. Both sensors collect several parameters, but we only focus on the data which are collected by the standard for medical ICU monitoring (Nihon Kohden USA; Irvine, CA, USA). The vital signs this study focuses on include heart rate, respiratory rate, and peripheral capillary oxygen saturation, as these can be collected by both types of biosensors and the Nihon Kohden monitoring system.

The study was conducted in an eight-bed medical ICU. The staff consists of an attending pulmonologist, one advanced practice provider, and four to five nurses. They were introduced to the study and hardware during a brief 10-min orientation. Patients were approached for consent while being in the ICU. Seven individuals agreed to participate, while one refused. One individual wore two sensors subsequently. The demographic characteristics of the study subjects are detailed in Table 1. After consenting, a patient was fitted with a sensor using the respective manufacturer’s recommendation. The staff was instructed to keep the sensor on for a 24-h period. After the collection of data, the sensor was removed. Patients and staff members were asked to complete a quick survey in the RedCap database (Appendix A.1) [18]. In addition, we asked the staff to complete a separate survey after the trial period to explore their perception of biosensors (Appendix A.2).

The data obtained from the biosensors were analyzed and compared to standard clinical monitoring provided using correlation and pathway analysis. Parametric variables were expressed as mean ± SD and compared using a Student’s *t*-test. For non-parametric variables, median (Me) and interquartile ranges (IR) were computed. Mann–Whitney U statistics were employed to compare non-parametric variables. Data groups were analyzed as independent groups. A double-sided p-value of less than 0.05 was considered statistically significant for all tests. The r-Pearson statistic was calculated to determine the correlation between the studied variables. Statistical analyses were performed using Statistica 11.0 (StatSoft Inc., Tulsa, OK, USA). Graphs were generated using GraphPad Prism 8.4.2 (GraphPad Software Inc., San Diego, CA, USA).

## 3. Results

### 3.1. Data Accuracy

**Figure 1:**
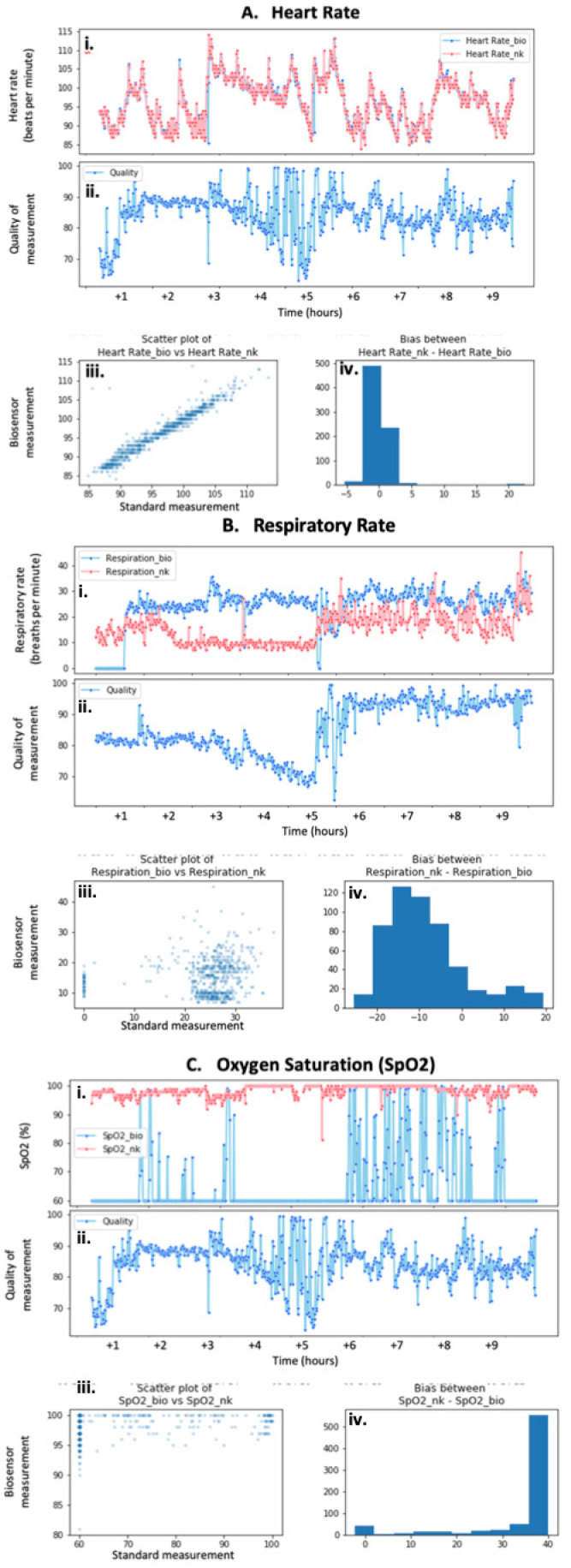
Correlation between data supplanted by multimodal sensor and standard ICU monitoring. Various degrees of data consistency were demonstrated by biosensors ranging from excellent for heart rate measurements (**A**), to variable for respiratory rate observations (**B**), to suboptimal SpO_2_ recordings (**C**). In addition to the vital signs measured (**i**) and the quality of the biosensor measurements (**ii**), the correlation (**iii**), and bias (**iv**) between biosensor and Nihon Kohden recordings were also reported according to vital sign.

The biosensors’ data showed varied performances with respect to different vital signs. Compared to respiratory rate and peripheral capillary oxygen saturation (SpO_2_), heart rate measurements demonstrated the strongest and most consistent correlation between a biosensor and wired ICU standard recordings at rest (Figure 1A(i),(iii)) and during movement (data not shown). Although the quality of the heart rate data fluctuated throughout this specific trial, it remained above 80% for most of the measurements recorded after the application of the biosensor (Figure 1A(ii)). The difference between the biosensor’s recordings and those of the Nihon Kohden system is assessed as the bias of the measurements, which is minimal and optimal for heart rate readings (Figure 1A(iv)). However, one trial demonstrated a significant lapse in the correlation during the onset of the measurements. The quality of the biosensor’s measurements during this time was significantly less than once the heart rate stabilized.

The correlation between biosensor and monitor-driven measurements for respiratory rate was significantly more variable than that of the heart rate recordings. One sample displayed superficially close correlations with similar results for both the biosensors and the manual measurements (Figure 1B(i)). This was confirmed by the weak positive relationship seen on the scatter plot that described the correlation between the two types of measurements (Figure 1B(iii)). Similar to the heart rate sample previously discussed, the quality of the measurements fluctuated throughout the trial, especially in the first half (Figure 1B(ii)). The bias reporting the difference between the biosensor and Nihon Kohden respiratory rates is visibly more than that seen for the heart rate data, further emphasizing the increased variability between the two forms of recording (Figure 1B(iv)).

The SpO_2_ measurements showed the most variability in terms of the correlation between the biosensor and standard monitor measurements. Most samples reflected no SpO_2_ measurements on the biosensors’ parts (Figure 1C(i)). This lack of recording was seen in at least three different samples. Interestingly, the evaluation of the biosensors’ quality did not reflect this, and instead remained at above 80% for the majority of the trial (Figure 1C(ii)). On another occasion, the biosensor only recorded periodically and at various qualities (data not shown). Similarly, the corresponding scatterplot for this sample does not reflect any correlation between the types of measurements (Figure 1C(iii)). The difference between the biosensor and standard monitor recordings seems to be greater than that of the heart rate measurements, as supported by the bias diagram (Figure 1C(iv)).

### 3.2. Deployment of the Sensors

The perspectives of patients wearing the biosensors, providers wearing the biosensors (providers as subject), and providers applying the biosensors on patients (provider for patients) were obtained through questionnaires to gauge the operationalization and ease of implementation of the biosensors. Determining the form factor and acceptance related to the biosensors is critical because these factors drive the discussion on implementation using the perspectives of both patients and providers. Specifically assessing the viewpoint of providers wearing the biosensors serves as an interesting comparison in relation to that of the patients they are treating.

**Figure 2:**
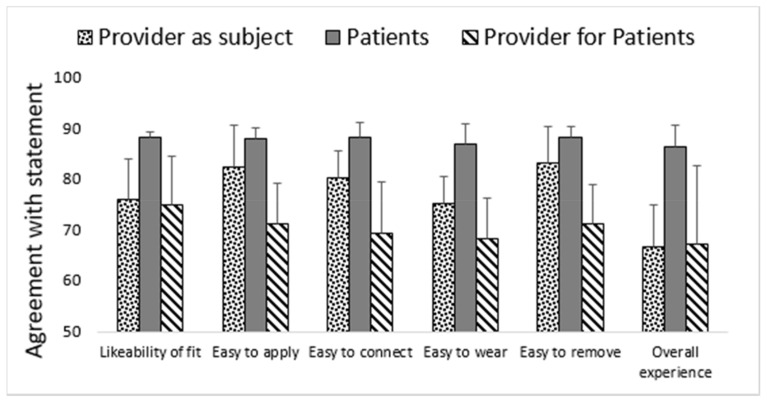
Experience of wearing the sensor. Experience of wearing the sensor was consistently rated higher for patient users compared to providers involved in care of patients.

The devices’ adherence to the skin was perceived as somewhat problematic by healthy individuals. Despite small form factor, most of the users and medical staff considered sensors to interfere with daily activities (Figure 2). Medical staff included MDs (medical doctors) and RN (registered nurses). Irritation was reported by a minority of the patients, with one individual reporting skin abrasion out of a total of eight patient trials (Figure 2). Only one trial was terminated before the prescheduled time because of the irritation. The operationalization of the sensor was assessed very highly by patients wearing them when asked how much they agreed with the following: “Did you like the way the biosensor fit?”, “Was it easy to apply?”, “Was it easy to connect?”, “Was it easy to remove?”, and “How was your overall experience related to biosensor?” (Figure 2). Finally, the sensor trials were terminated on time, at the prescheduled time, in all study groups (providers as subjects = 65%, patients = 75%, providers for patients = 92%). Neither of the clinical groups discontinued the sensor because of interference with clinical care.

### 3.3. Perception of the Sensors

**Figure 3:**
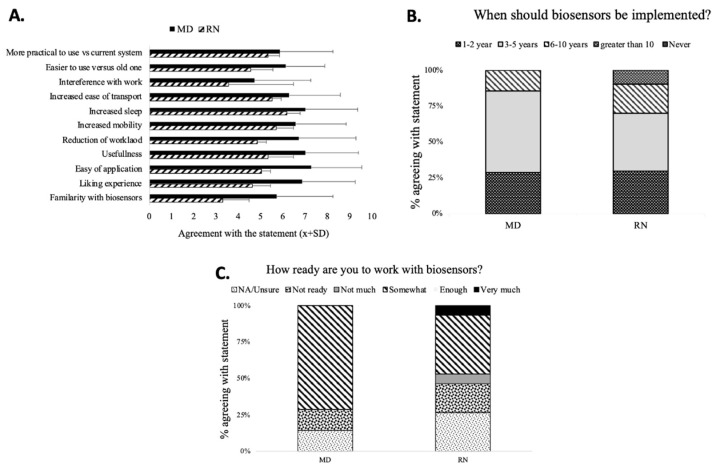
Readiness for implementation of biosensors. Physicians assessed the benefits of sensor deployment highly (**A**) and predicted faster implementation (**B**) than nurses. Nurses reported a more slightly unprepared perception of readiness to work with biosensors (**C**).

There was little difference in perception of the different domains of the sensors’ usability between MDs and RNs, except for the familiarity with sensors between RNs and MDs (Figure 3A). The most common positive comments about sensors were “modern/sleek”, “mobility”, and “more data”. The most common negative adjectives were “application”, “unreliable”, and “cost”. The major sensor advantages were “easy application”, “not-obstructive”, and “portability”.

The majority of MDs and RNs believed that sensors would be deployed in the next 3 to 5 years (B). The staff was feeling relatively unprepared for sensor deployment (Figure 3C).

## 4. Discussion

The implementation of biosensors demonstrated several important related problems. The reliability of a sensor has to be extensively studied before the implementation. Prior reports pointed to unique problems related to the biosensors, although this was not the uniform case [12,16,19,20]. Movement, skin color, and sweating were quite often reported as the main reasons for interference [12,19]. Post-deployment interviews demonstrated that data might be lost for other reasons [14]. Sensor adherence was cited as such, but the loss of some data could not be explained exclusively. Considering that the correlations between the biosensor and clinical recordings for respiratory rate and SpO_2_ were not significantly accurate, the variability that was introduced could negate the reliability and accuracy of the biosensors [21,22,23,24].

Overall, data correlation depended more on the data type (e.g., vital sign recorded) than on the sensor type in our study, and that was a new finding [12,16,19]. The weak correlations between readouts of the sensor and clinical standards augment the skepticism regarding integrating the biosensors with more standard critical care technology. Without a standard for accuracy, the variability will require consistent validation of the results, which will be both time sensitive and concerning if the validation fails. These problems emerged even before we could test the sensors’ connection to the IT system. The unpredictability of the biosensors connecting to the appropriate downloading devices or tablets is one of the main concerns regarding this novel technology [4]. Being unable to anticipate if or where the biosensor will connect is one possible restriction that diminishes the fidelity of biosensors, given they should function to wirelessly monitor patients at all times. The stability and resiliency, among other technological obstacles, of electrochemical biosensors have proven to be focal points for barriers to their implementation, and the acceptance rate for loss signal has not being established [10]. However, our study demonstrated that multi-sensor devices might be uniquely prone to sensing errors as compared to clinical standards. This is a new and unique finding [12,16].

The adverse effects of wearing the sensors were rare. Irritation was almost not observed, while only one case of abrasion was noted in our study. The small number of enrolled subjects precluded this from being a conclusive study. Future studies should look into the incidence of adverse effects related to biosensors’ application as compared to regular monitoring. However, most of the devices are fairly inert while being worn by patients [6,10,12,16].

The acceptance of the biosensor technology was particularly high for patients and slightly less so among the providers. This was the novel finding of the study, since some reported several barriers [14,16]. The reason driving the high acceptance of the biosensors was the relatively low form factor of devices [4,21,23]. A desire for non-interference of the device was frequently cited [16]. We demonstrated relatively low initial enthusiasm at the beginning of the trial that significantly increased at the completion of trials. Patients had overall positive impressions. The interference with workflow was minimal, though providers wearing the sensors reported much higher rates of premature termination of the trials secondary to adherence problems. The increased mobility of healthy individuals compared to bedridden patients may be partially responsible for this difference [14,16].

Our study has several limitations. This was not a device trial, or even a pilot study. The sample size was small, and we used two different devices. Devices were placed on few patients or staff members. However, the intention of this paper was to observe the implementation process to demonstrate potential problems. Much too often, the problems during implementation are not brought up, setting unrealistic expectations from the end-user.

## 5. Conclusions

We caution against an overoptimistic approach to the implementation of biosensors in a healthcare setting, as the process has several potential pitfalls. Despite being FDA- approved, biosensors need to be consistently tested against standard monitoring equipment, such as that of Nihon Kohden, in order to demonstrate readiness for implementation in high-acuity healthcare settings.

## Figures and Tables

**Table 1:** Demographic characteristics of studied cohorts.

**Patients *n* = 8**		
Age (x ± SD)		59 ± 9
Sex	M	2
	F	6
Race	Caucasian	4
	Asian	1
	African American	3
How long being worn	1–4 h	0
	5–24 h	8
	1 day to 1 week	0
**Providers taking care of patients *n* = 13**		
How long being worn	1–4 h	0
	5–24 h	10
	1 day to 1 week	3
Profession	MD	8
	RN	5
**Providers wearing devices *n* = 16**		
How long being worn	1–4 h	8
	5–24 h	7
	1 day to 1 week	1

## Data Availability

The datasets used and/or analyzed during the current study are available from the corresponding authors on reasonable request.

## Appendix A

### Appendix A.1. The Questionnaire Used to Study the Attitude of the Staff towards Biosensors in Patients and Staff

1. What is your role?
  - Attending
  - APP
  - RN
  - CNA
  - Other staff
2. Did you have contact with biosensor before
  - Y/N
3. How familiar are you with biosensors (0—not at all; 10—extremely familiar)
  - 0–10
4. Did you like the experience? (0—not at all; 10—extremely familiar)
5. Is the biosensor easily applied to the participants? Y/N
6. Do you think they are useful? (0—not at all; 10—extremely)
7. How much biosensor can potentially alleviate your workload? (0—not at all; 10—extremely)
8. Do you think patients like them? (0—not at all; 10—extremely)
9. How do you think it will impact the patient’s experience
  - Mobility (0—not at all; 10—extremely)
  - sleep (0—not at all; 10—extremely)
  - transport? (0—not at all; 10—extremely)
10. How much did they impair on the work? (0—not at all; 10—very)
11. Compared to current monitoring equipment:
  - How esay is the biosensor to use (0—not at all; 10—very)
  - How practical Is the biosensor to use in the healthcare setting (0—not at all; 10—very)
12. When they should be implemented?
  - Never
  - 1–2 years
  - 5–6 years
  - In 10 years
  - Never
13. What is the main advantage of biosensor?
14. What is the major problem with biosensors?
15. List up to three adjectives describing this device?
  - X
  - X
  - X
16. How familiar is the staff with devices after the trial (0—not at all; 10—very)
17. How ready is staff for their implementation? (0—not at all; 10—very)

### Appendix A.2. The Questionnaire Used to Study the Attitude of the Staff towards Biosensors in Patients and Staff

1. What is your role
  - Attending
  - APP
  - RN
  - CNA
  - Other staff
2. Did you have contact with biosensor before
  - Y/N
3. How familiar are you with biosensors
  - 0–10
4. Did you like the experience?
5. Do you think they are usefull
6. Do you think patients like them
7. How much did they impair on the work?
8. Do you think
9. When they should be implemented
  - Never
  - 1–2 years
  - 5–6 years
  - In 10 years
  - Never
10. What is the main advantage for biosensor
11. What is the major problem with biosensors
12. List up to three adjective describing this device?
  - X
  - X
  - X
13. How familiar is the staff with devices
14. How ready is staff for their implementation?
